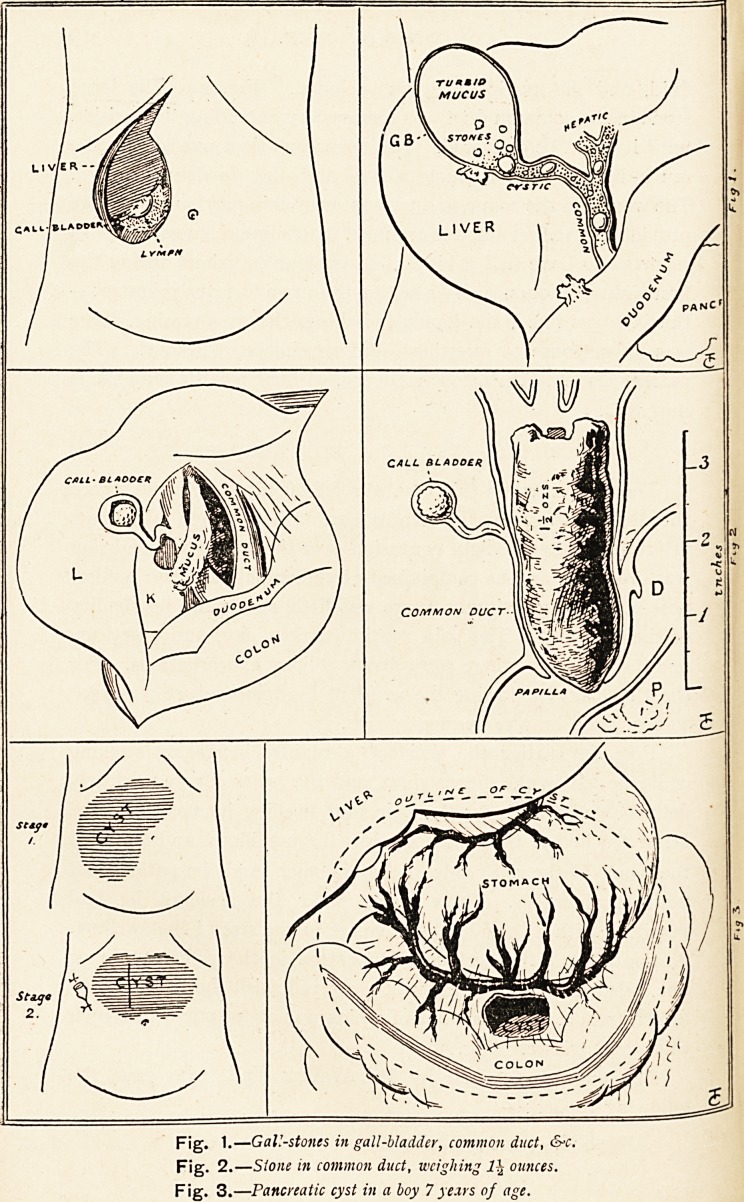# Notes on Cases
1Shown at the meeting of the Bath and Bristol Branch of the British Medical Association, Oct. 28th.


**Published:** 1908-12

**Authors:** T. Carwardine

**Affiliations:** Surgeon, Bristol Royal Infirmary


### NOTES ON CASES.[1]

BY

T. CARWARDINE, M.S.,
*Surgeon, Bristol Royal Infirmary.*

### GALL-STONES IN GALL-BLADDER, CYSTIC DUCT, COMMON DUCT, AND HEPATIC DUCTS, IN A GIRL OF SEVENTEEN.

THIS case is noteworthy from the youthful age of the patient, and the extent of involvement of the biliary apparatus.

She had indigestion two years previously, independent of food. Three months ago she had pain across the stomach and back, chiefly on the left side, and jaundice appeared when the pain ceased. A short time ago she had return of severe pain beginning and ceasing suddenly, lasting several days, and followed by jaundice. Thus she had two attacks of pain recently, in the first of which the urine was dark and the motion light.

[1] Shown at the meeting of the Bath and Bristol Branch of the British Medical Association, Oct. 28th.

When admitted she had a little jaundice and no other symptoms.

At the operation there were adhesions about the gall-bladder, which was surrounded by jelly-like lymph. (Fig. 1.) The liver was large and congested; and the gall-bladder, of yellowish colour, contained a milky fluid, stones and sand. On exposure of the ducts they were all found to be hard from contained stones. An incision was made into the common duct, where there was a large stone, and it was extended up into the cystic and common hepatic ducts, which were full of stones and grit. After these were cleared out more debris was found in the right and left hepatic ducts, which were also cleared. The pancreas was hard. Adequate drainage was provided, and the patient made a perfect recovery, leaving three weeks after operation.

### STONE IN COMMON BILE DUCT WEIGHING 1½ OZ., AND MEASURING 3½ INCHES LONG AND 4 INCHES IN CIRCUMFERENCE.

This is an example of an enormous gall-stone removed from the common duct during life; although there is recorded an example of a larger stone, which weighed nearly 3½ oz., removed from a patient after death. In the latter case there is evidence that bile passed into the duodenum, for it is recorded that fluid bile surrounded the stone; and in my case, although the stone was so large, and its lower end fitted like a ball-valve into the dilated ampulla of Vater, the patient was not deeply jaundiced.

She was a woman of 64, who had been subject to bilious colic for 37 years, and on two occasions gall-stones had been found in the stools. She was admitted a week after a bad attack of colic with rigors and sickness; the jaundice cleared up but again increased, and a hard resistant mass could be felt below the right hypochondrium, below which the lower border of the liver could be felt some few inches.

When the abdomen was opened a very hard mass presented itself in the common duct, which gave a ringing sound when percussed with a pair of forceps. The gall-bladder was very small, and contained a solitary calculus surrounded by a little turbid fluid, and the cystic duct was occluded. (Fig. 2.) The large stone in the common duct was removed by extending the incision until it could be delivered. The finger was then passed into the much-dilated hepatic ducts above and the duodenum below. The wound in the common duct was sutured in part, and drainage provided. At first she was troubled with some sickness, and was reported to have had a kind of fit on four occasions during the first eighteen hours, each fit lasting from two to seven minutes :— the jaw was fixed, the hands twitching, the eyes staring, there was no response to questions, and no change of colour. The patient made a satisfactory recovery, without pyrexia, and she is now well.

PANCREATIC CYST IN A BOY AGED SEVEN.

The patient was a thin boy, who fell off his bicycle and struck the abdomen against the handle bar. He was seen two hours afterwards, when a slight contusion over the left hypochondrium was observed, and he complained of abdominal pain and tenderness in the epigastrium. There was little shock and no rigidity of the abdomen. The pain continued next day, and every ten or fifteen minutes there were severe colicky exacerbations. For the three days following the accident his temperature was 100° to 101°, and he was frequently sick.

Five days after the accident a rapidly increasing swelling could be felt in the epigastrium, and the lower edge was clearly defined at first. Later the pain and swelling increased till the latter reached to three inches below the umbilicus, and occupied the whole of the right side of the abdomen, and the patient lost flesh considerably. Three weeks after the accident he had vomiting with a considerable increase in the size of the swelling. A month after the accident, I operated in the right semilunar line, when about three pints of clear yellowish fluid were withdrawn from a cyst, the wall of which was about one-eighth inch thick and friable. (Fig. 3.)

The fluid was tested by Dr. Walker Hall, who gave the following report :—

S.G.—1008 ; alkaline.

Fig. 1.—*Gall-stones in gall-bladder, common duct, &c.*

Fig. 2.—*Stone in common duct, weighing 1½ ounces.*

Fig. 3.—*Pancreatic cyst in a boy 7 years of age.*

Albumin—5 parts per 1000 Esbach.

Fat—Large traces in ether extract.

Deposit—Numerous cells containing fat droplets. Small amount of pigment, cholesterin and calcium crystals.

*Digestive Action.*—After forty-eight hours there is distinct evidence of the digestion of protein. (Trypsin therefore present.)

*Reducing Substance.*—There is present a reducing substance which is not sugar.

After the operation very little more fluid escaped, and four days afterwards a swelling appeared in the epigastrium, rapidly increasing in size, and pushing the original drainage-tube downwards and outwards in the right loin. The colicky pains returned and the patient looked more ill. A second operation was, therefore, performed six days after the first through the right rectus muscle. The stomach was immediately below the incision, and adhesions to the parietes were separated. An opening through the gastro-colic omentum revealed a tense cyst, from which about one and a half pints of fluid, with some coagulated serum, escaped. The discharge was continuous and profuse; caused considerable irritation of the skin, as if from digestion; and the patient continued to waste. The cavity was, therefore, washed out with weak adrenalin solution, and the patient was fed with raw and cooked sweetbread. After ten days the discharge rapidly diminished, the wound soon healed, and the patient is now perfectly well, a year after the operation.

This case is a well-marked example of false pancreatic cyst described by Mr. Jordan Lloyd in 1892, resulting from injuries to the pancreas, and it bears out the contention that the lesser peritoneal cavity tends to be involved. Such cysts have occurred between the ages of thirteen months and seventy-six years, and as they arise from the pancreas in the retro-peritoneal region, they are covered by three layers of peritoneum. In the case here recorded there is distinct evidence that the cyst first occupied the lesser peritoneal sac, and then involved the right loin through the foramen of Winslow. After the first operation the foramen of Winslow must have become closed, leaving a retention cyst in the lesser sac of peritoneum, which required subsequent drainage. This view is confirmed by the fact that Albert saw a bulging of such a cyst through the foramen of Winslow. The mortality of the recorded cases has been about 10 per cent.; and it will be interesting to observe whether my patient develops diabetes in the future.

## Figures and Tables

**Fig. 1. Fig. 2. Fig. 3. f1:**